# Preparing Children for Their First Dental Visit: A Guide for Parents

**DOI:** 10.3390/healthcare10112321

**Published:** 2022-11-19

**Authors:** Simone Bagattoni, Francesca Nascimben, Elena Biondi, Raquel Fitzgibbon, Lisa Lardani, Maria Rosaria Gatto, Gabriela Piana, Katia Mattarozzi

**Affiliations:** 1 Unit of Special Needs Dentistry and Paediatric Dentistry, Department of Biomedical and Neuromotor Sciences, University of Bologna, 40125 Bologna, Italy; 2 Unit of Pediatric Dentistry, Department of Surgical, Medical and Molecular Pathology and Critical Care Medicine, University of Pisa, 56126 Pisa, Italy; 3 Department of Experimental, Diagnostic and Specialty Medicine, University of Bologna, 40126 Bologna, Italy

**Keywords:** pediatric dentistry, behavior management, dental anxiety

## Abstract

The aim of the study was to test an information booklet containing suggestions to parents on how to prepare their child for the first dental visit. Forty-five children and one parent per included child took part in the trial. Children were randomized in two groups; the information booklet was e-mailed to the parents of the study group. At the end of the visit, the dentist and the parent evaluated the child’s behavior through the Frankl Behavior Rating Scale (FBRS) and the utility of the booklet through a Likert scale. The children evaluated the pleasantness of the visit and the perceived pain through the Wong–Baker FACES^®^ Pain Rating Scale (WBFPRS). Parents evaluated the information booklet as highly understandable and useful. According to the dentist, informed children were more cooperative (FBRS median score: 4; IQR: 3.5–4) than the control group (median score 3; IQR: 2–4) (*p* = 0.013; Mann–Whitney U test). Children prepared with the booklet reported less pain (WBFPRS: 0.40 ± 0.82 vs. 1.42 ± 1.99; *p* = 0.034; *t*-test;) and tended to evaluate the visit as more enjoyable (WBFPRS: 1.1 ± 2.14 vs. 2.75 ± 3.43; *p* = 0.064; *t*-test) than unprepared children. The information booklet increases the child’s ability to cooperate during the visit and could represent a useful instrument for the clinical practice.

## 1. Introduction

In medicine and dentistry, the treatment’s effectiveness derives not only from the competence of the physician but also from the ability to create an effective relationship with the patient. When the patient is a child or a special-needs patient, the relationship is more complex [1,2]. The dentist has a dual task: to deal with the child’s possible resistance arising from fear of the unknown and potentially threat; to deal with parents’ behavior, often unprepared to adequately guide their child toward dental care [3]. Obtaining the child’s and parents’ cooperation, while promoting a positive attitude toward dental care, is a primary goal for the pediatric dentist [4].

In this context, preoperative communication is very useful, but often underestimated. Many studies have shown how the information children receive about the dental environment before the visit can influence their behavior, both positively and negatively. A previous study showed that presenting pictures of children enjoying the dental visit promotes a positive relationship with the dentist [5]. The study showed a reduction in anticipatory anxiety, the unpleasant sensation that afflicts children during their first dental experience [5]. The study by Melamed et al. [6] showed that children that were previously prepared for restorative procedures watching a video of a peer undergoing the same procedure can overcome their fears and be more cooperative. The children of the control group, who were shown a video with nondental content, reported a higher level of anxiety.

Even though the child acquires information of the therapy’s steps and instruments, they have never experienced them directly; thus, they can be frightened of what they will feel. It is, therefore, necessary to provide preparatory sensory information; this helps the child to cope with fear and pain, especially when combined with the use of distraction. Distraction alone may not be enough if the child, unaware of what will come, is tense and unable to distract themself; the prepared child knows what awaits them and is more easily distracted [7,8]. As shown in several studies, the parent’s state of anxiety greatly influences that of the child; hence, the preparatory information is also useful for reassuring the parent [9].

To date, not enough attention has been paid to the role of communication before the first dental visit to encourage the child’s cooperation and avoid dysfunctional behaviors. The dentist can provide parents with the information they need to adequately prepare the child for treatment. The present study aimed to investigate the effectiveness of an information booklet to promote pediatric patients’ cooperation during the first dental visit. We hypothesized that offering guidance to parents on how to prepare their children for the first visit would (I) reduce the degree of unpleasantness of the visit for children, (II) reduce the child’s pain perception, (III) increase the child’s cooperation, and (IV) be appreciated by parents.

## 2. Materials and Methods

This was a single-center parallel-group study. We adopted a single-center approach to guarantee consistency regarding equipment, environment, and data collection. The study took place at the Unit of Pediatric Dentistry of the Department of Biomedical and Neuromotor Sciences of the University of Bologna.

The study design was approved by the Ethics Committee of Area Vasta Emilia Centro (CEAVEC) on 23 January 2019 (protocol No. 0033664, ref 69/2019/SPER/AUSLBO) and registered on ClinicalTrial.gov (NCT05608720).

### 2.1. Sample Size

Preliminary results from a pilot study carried out by the same scientific committee (not published data) evidenced an average of visit pleasantness rated by children equal to 0.9 in the study group and 2.7 in the control group. Consequently, at an alpha level equal to 0.05 with a power of 80% for a two-sided test and an allocation ratio of 1:1 between the two groups, a sample size of at least 21 children was needed in each group. 

### 2.2. Recruitment and Randomization

Eligible participants were parents and their children who made an appointment for a first visit at the Unit of Pediatric Dentistry of the Department of Biomedical and Neuromotor Sciences of the University of Bologna between January 2019 and September 2019. The parents of 158 children were initially contacted by telephone by the principal investigator, a pediatric dentist, to check the exclusion/inclusion criteria and to obtain a preliminary verbal informed consent for study participation. A total of 102 children were excluded because they met one or more of the exclusion criteria (i.e., previous dental visits, intellectual disability, and mother tongue other than Italian). Parents were then emailed information regarding the objective of the study to obtain formal informed consent to participation. Of the 56 eligible children, eight did not show up on the day of the visit, and the parents of three children did not consent to participation in the study. Forty-five children and one parent each were included in the study. After obtaining informal consent by e-mail, participant randomization was performed. Each participant was assigned an alphanumeric identification code. The parent and respective child were identified with the same number and a different letter code (example: child C_01, mother M_01, and father F_01). The participants were randomly assigned to the study or control group.

### 2.3. Procedure

Two days before the dental visit, the parents within the study group received the information booklet as a PDF file by e-mail. The parents and children in the control group received the usual information (i.e., day, time, place of the appointment, and bureaucratic information) along with a thank you for participating in the study.

The day of the dental visit, written informed consent was collected, and the children of both groups were visited by a pediatric dentist on duty at the time, blinded to patient group allocation. The first dental visit consisted of a visual examination of the oral cavity with the aid of a dental mirror and a dental probe to assess the oral health status of the child. A tell–show–do approach was utilized. No operative procedures (e.g., fillings or X-rays) were performed. At the end of the dental visit, the principal investigator handed the pediatric dentist, the parent, and the child a paper questionnaire.

**Figure 1:**
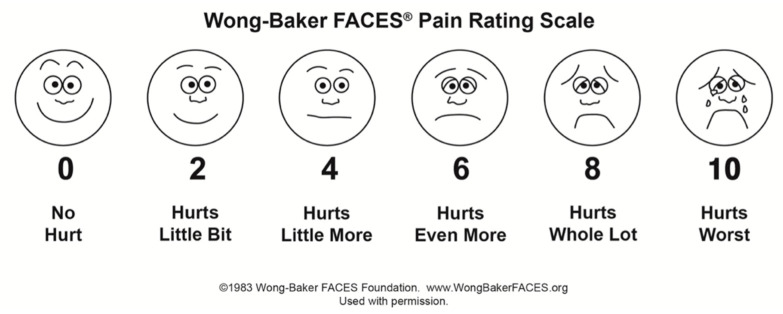
Wong–Baker FACES Pain Rating Scale.

The child’s questionnaire investigated the pleasantness of the visit and the perceived pain using the Wong–Baker FACES Pain Rating Scale (WBFPRS) with corresponding scores from 0 to 10 [10] (Figure 1).

The dentist’s questionnaire investigated the child’s behavior during the visit using the Frankl Behavior Rating Scale (FBRS) [11] (1 = definitively negative, 2 = negative, 3 = positive, and 4 = definitively positive).

The parent’s questionnaire investigated the behavior of the child during the dental visit through the FBRS; if part of the study group, it also investigated the evaluation of clarity, comprehensibility, usefulness, ease of application, and truthfulness of the booklet through the Likert scale to five points (1 = very little, 5 = very much).

### 2.4. Information Booklet Description

**Figure 2:**
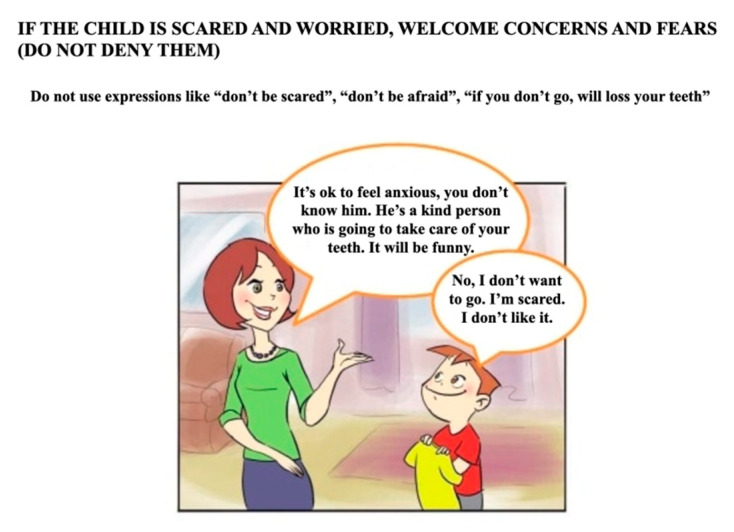
Example of easy-to-understand language and colorful images adopted.

The information booklet was ad hoc written on the basis of the effectiveness of communication in the doctor–patient relationship [12]. The choice of concepts and words was based on the literature on stress and the nocebo effect [13]. The objectives were to increase knowledge about the first dental visit and to prevent the child from activating negative expectations and aggressive or avoidance responses. The booklet was written in Italian, and it consisted of four pages with texts and pictures showing a parent and a child talking about the visit. Specifically, the first part of the booklet explained to parents the importance of the first dental visit in promoting a positive attitude toward the dental environment. The second part suggested how the parent should prepare the child for the visit: “inform the child about the visit to the gentle dentist using truthful and positive words”; “accept the child’s fears and concerns without denying them”, using phrases such as “you must not be afraid”; “prepare the child for some simple procedures, such as ‘sit down, it is time to count your teeth’”; “welcome the concern and fear expressed by the child”; “propose a cartoon about Peppa Pig’s first dental experience (https://www.youtube.com/watch?v=xLN0smEFoPI, Peppa Pig episode 2 × 37 ‘At the Dentist’, accessed on 15 December 2018)”; “do not use words with negative emotional valence”; “do not promise that unpleasant events will not occur”; “do not promise gifts”; “do not talk about negative dental experiences” (Figure 2). Easy-to-understand language and colorful images consistent with written information were used to make communication more effective. The booklet is freely available upon request to the corresponding author.

### 2.5. Statistical Analysis

Statistical analysis was conducted with SPSS software (27.0 version, SPSS Inc., Chicago, IL, USA). A Kolmogorov–Smirnov test verified the Gaussian distribution of the variables. Consequently, the mean and standard deviation were calculated for WBFPRS scores; the median and interquartile range (IQR) were calculated for FBRS scores. The *t*-test and Mann–Whitney U test were used for the comparison of continuous variables and the chi-square test was used for categorical variables. The significance level was set at *p* < 0.05. The biostatistics were masked to the group allocation.

## 3. Results

### 3.1. Sample

Twenty-one children were included in the study group, along with 24 in the control group. Descriptive characteristics of children, parents, and operators are described in Table 1. No significant differences were found between the two groups.

### 3.2. Information Booklet

The average scores provided by the parents concerning clarity, comprehensibility, usefulness, ease of application, and truthfulness of the booklet are shown in detail in Table 2.

**Figure 3:**
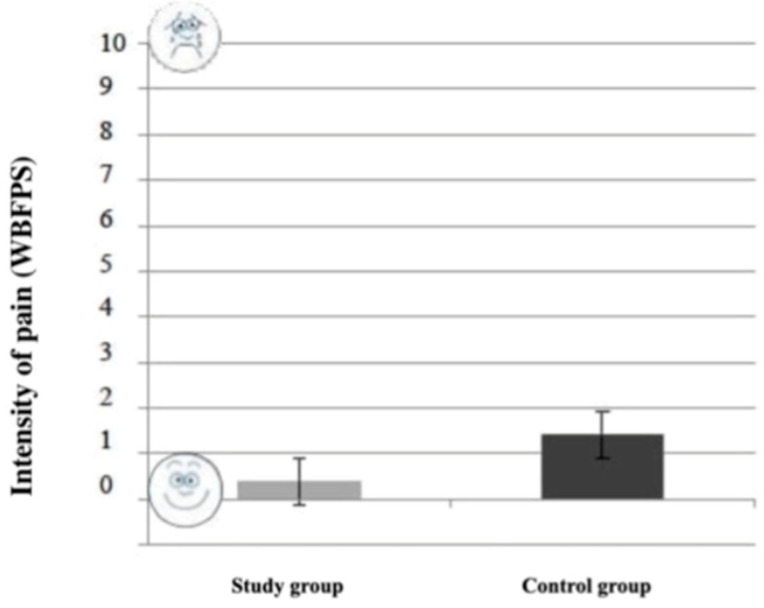
Reported pain by children in the two groups.

The difference in pain reported by the children during the visit between the study group (0.40 ± 0.82) and control group (1.42 ± 1.99) was statistically significant (*p* = 0.034; *t*-test). As shown in Figure 3, children in the study group reported less pain than the control group.

**Figure 4:**
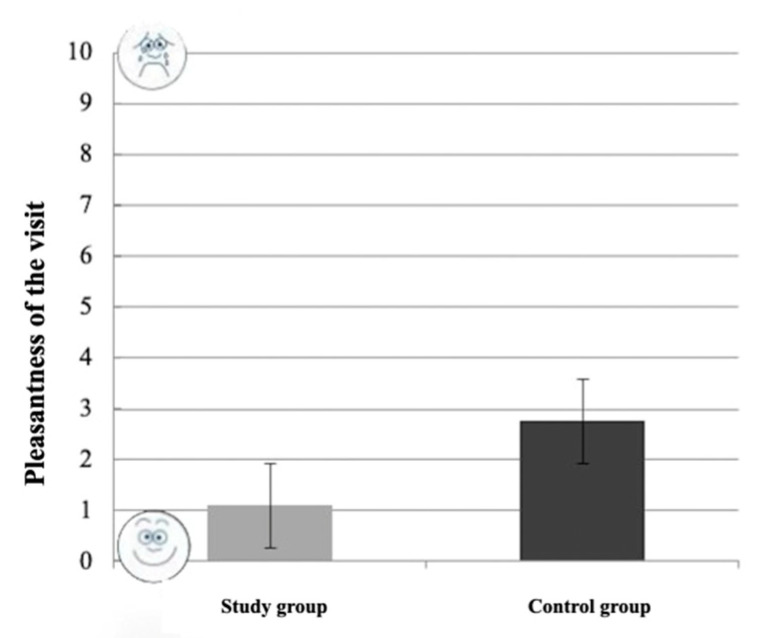
Reported pleasantness of the visit by children in the two groups.

The difference in pleasantness reported by the children during the visit between the study group (1.1 ± 2.14) and the control group (2.75 ± 3.43) was not statistically significant (*p* = 0.064; *t*-test). As shown in Figure 4, children in the study group tended to evaluate the visit as more pleasant than the control group (lower scores indicate higher approval).

**Figure 5:**
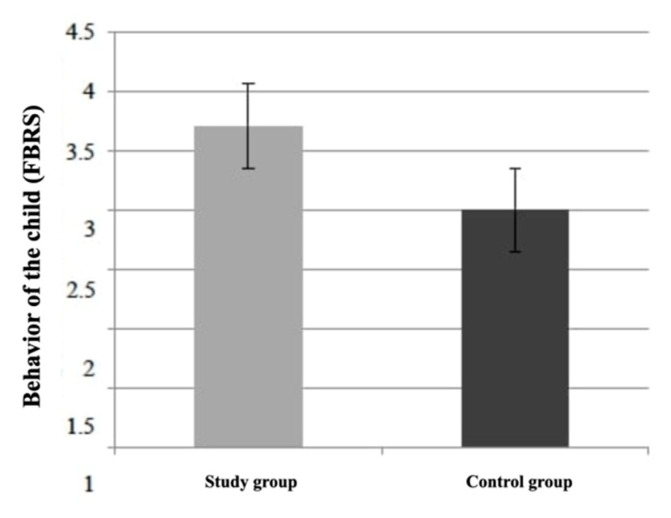
Dentists ‘evaluation of children’s behavior.

The difference in children’s behavior assessed by the operators between the study group (median: 4; IQR: 3.5–4) and the control group (median: 3; IQR: 2–4) was statistically significant (*p* = 0.013; Mann–Whitney U test). As shown in Figure 5, operators judged the children in the study group as more cooperative compared to the control group.

The difference in children’s behavior assessed by parents between the study group (median: 4; IQR: 3–4) and the control group (median: 4; IQR: 2.25–4) was not statistically significant (*p* = 0.347; Mann–Whitney U test).

## 4. Discussion

Negative, painful, and invasive experiences play a central role in the etiology of dental fear, and they often date back to childhood and adolescence. Children who had a negative experience since the first approach to the dentist have a higher risk of suffering from dental fear than children who had only a negative or painful experience after several positive experiences [14,15]. Hence, this shows the importance of setting up a structured first dental visit that activates positive experiences and nontraumatic memories.

This is the first study in the literature to focus on the child’s preparation before the first dental visit. This preparation took place in a safe environment and by an affectively relevant person such as a parent informed by the booklet. Parents appreciated the information booklet considering it clear, easy to use, and truthful. Our results show that guiding parents to prepare their child for the first dental visit has a reassuring effect on both sides. Parents felt involved in the process from the beginning and felt ready to explain the situation to their child. Children showed a more cooperative behavior, and they tended to find the visit more enjoyable than the children in the control group. Notably, they felt less pain during the procedure. For clarity, the first dental visit in both groups did not include operative or invasive procedures. The reported pain is probably an expression of the stress experienced during the visit. However, the lower reported “pain” of the study group could be explained by less anticipatory anxiety and an increased sense of control toward an unknown experience [16]. Many studies agreed in identifying negative experiences and anxiety as powerful modulators of pain perception, including in dentistry [17,18]. However, the relationship between anxiety and preparation may have a twofold trend; too much or too little information can increase anxiety levels [19]. Therefore, it is important to pay attention to the quality and quantity of information; the booklet provides the parent with specific indications on what to say and what not to say to the child [20].

An important result emerged from the pediatric dentists’ evaluation: children in the study group were more cooperative than the unprepared children. This outcome represents a great advantage for the pediatric clinician and potentially even more so to the general practitioner. In contrast, parents did not see their children’s behavior as more cooperative. This could be explained by the fact that both groups were approached by experienced pediatric dentists, well trained in dental behavior management [21]. An increase in the number of participants could lead to a more consistent result, in accordance with the dentist’s assessment.

A limitation of the study concerned using single-item measures. However, we based our methodology on validated psychometric scales used to rate pain, emotional stimuli, and behavior during the visit. Randomization gave control over confounding variables that could not be held constant or measured, such as the reason for the first visit, the parents’ gender, age, and educational level, the parents’ personality traits and previous dental experiences, the child’s personality traits, or any other unknown confounding factors that could differentiate the group composition. Bias, potentially derived from the pediatric dentist who visited the child, was controlled by masking the procedure. Considering the aim of the study, masking of participants could not be applied. Data analysis potential bias was controlled by masking the biostatistics.

In accordance with the literature, our results confirm the importance of preparing parents and children for their first dental visit through booklets, simulation programs, or smartphone applications [22,23].

A further research project could evaluate the efficacy of the booklet in the long term (i.e., during dental treatment) and for children with previous negative dental experiences. In addition, it would be interesting to test the information booklet on general dental practitioners and to extend an adapted version to other care settings involving children. Lastly, a further evaluation of the difference between a written booklet and audiovisual material such as a video of a first visit could be tested.

## 5. Conclusions

Guiding parents to prepare children at home increases the ability to cope with the dental visit and decreases the perception of discomfort and pain. The information booklet is easy to implement in the clinical practice, both in private and in public facilities, and the cost is negligible.

## Figures and Tables

**Table 1:** Descriptive characteristics of the study and the control group.

	Study Group	Control Group
Children		
M	12	12
F	9	12
Age: years (±SD)	5.0 (±1.6)	5.8 (±2.3)
Parents		
M	4	4
F	17	20
Age: years (±SD)	39.2 (±6.7)	36.4 (±6.4)
Dentists		
M	4	3
F	3	4
Age: years (±SD)	33.1 (±6.1)	31.0 (±4.6)
Experience: months (±SD)	85.1 (±55.1)	72.8 (±41.6)

**Table 2:** Evaluation of booklet’s contents.

Informative Booklet Evaluation	Median [Interquartile Range]
Comprehensible and clear	5 [5–5]
Useful	5 [5–5]
Easy to do	5 [5–5]

## Data Availability

The data presented in this study and the information booklet are available on request from the corresponding author.
